# Progress on Polymer Dielectrics for Electrostatic Capacitors Application

**DOI:** 10.1002/advs.202202438

**Published:** 2022-08-18

**Authors:** Hang Luo, Fan Wang, Ru Guo, Dou Zhang, Guanghu He, Sheng Chen, Qing Wang

**Affiliations:** ^1^ State Key Laboratory of Powder Metallurgy Central South University Changsha Hunan Province 410083 China; ^2^ Key Laboratory of Polymeric Materials and Application Technology of Hunan Province College of Chemistry Xiangtan University Xiangtan Hunan Province 411105 China; ^3^ Department of Materials Science and Engineering The Pennsylvania State University University Park PA 16802 USA

**Keywords:** electrostatic capacitors, energy density, polymer dielectrics, relative permittivity

## Abstract

Polymer dielectrics are attracting increasing attention for electrical energy storage owing to their advantages of mechanical flexibility, corrosion resistance, facile processability, light weight, great reliability, and high operating voltages. However, the dielectric constants of most dielectric polymers are less than 10, which results in low energy densities and limits their applications in electrostatic capacitors for advanced electronics and electrical power systems. Therefore, intensive efforts have been placed on the development of high‐energy‐density polymer dielectrics. In this perspective, the most recent results on the all‐organic polymer dielectrics are summarized, including molecular structure design, polymer blends, and layered structured polymers. The challenges in the field and suggestions for future research on high‐energy‐density polymer dielectrics are also presented.

## Graphical abstract

The uniqueness of the review is mainly focused on all‐organic polymer dielectrics and aims to cover the molecular engineering strategies, including increased relatively permittivity, suppressed dielectric loss, enhanced breakdown strength, improved processability, and high‐temperature stability, in all‐organic polymer dielectric.

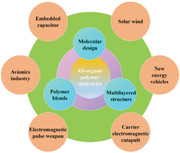

## 1 Introduction

**Figure 1:**
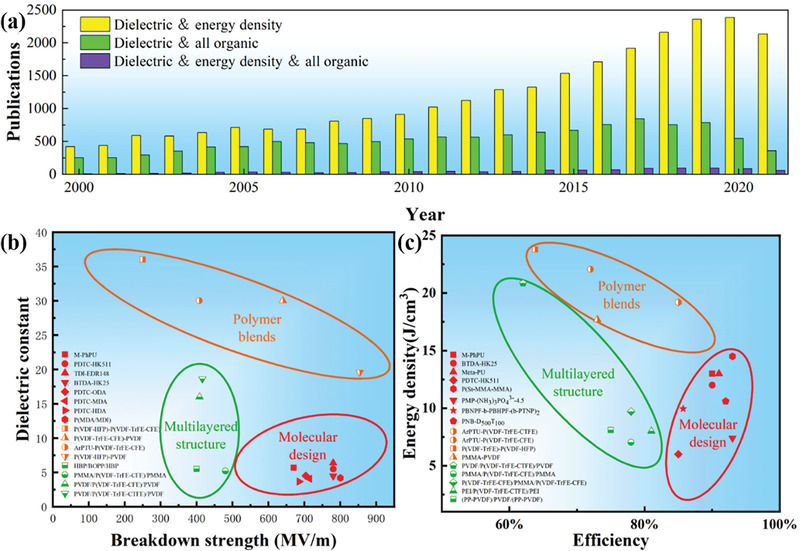
a) Increase of annual refereed publications dealing with “dielectric polymer”, “energy density”, and “all organic”; State‐of‐the‐art b) dielectric constant and breakdown strength, c) energy density and energy efficiency of various dielectric polymers.^[^ ^14^, ^15^ ^]^

**Figure 2:**
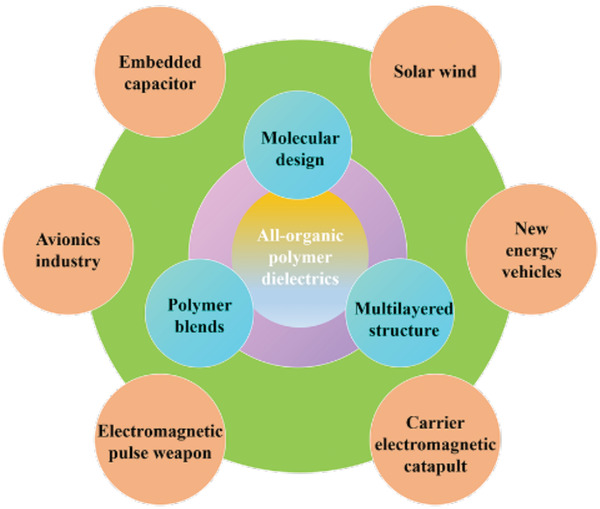
The emerging applications of dielectric capacitors

Electrostatic capacitor, also known as dielectric capacitor, is a kind of energy storage device, which is attracting interest in an increasing number of researchers due to their unique properties of ultrahigh power density (≈10^8^ W kg^−1^), fast charge/discharge speed (<1 µs), long life (≈500 000 cycles), high reliability and high operating voltage.^[^ ^1^ ^]^ In the past 10 years, the topic of dielectric capacitors is becoming a research frontier and hotspot. As shown in **Figure** **1**a, we counted publications related to the titles including the keywords of “dielectric polymer”, “energy density”, and “all organic” from 2000 to 2020. As can be seen, a quick growth number of papers focused on dielectric capacitors are published in these years. Due to a series of advantages summarized above, dielectric capacitors show a broad prospect employed in the areas of embedded capacitor, solar wind, new energy vehicles, carrier electromagnetic, electromagnetic pulse weapon, and avionics industry,^[^ ^2^ ^]^ presented in **Figure** **2**. As an example, the film capacitors are employed as DC support capacitor in new energy vehicle, such as Toyota PRIUS Hybrid, Tesla Model 3, and BYD Qin plus. Compared with aluminum electrolytic capacitors,^[^ ^1^, ^3^ ^]^ the film capacitors display self‐healing characteristics, higher better capability of withstand voltage, rated voltage, and long life.

Acting as the key factor in determining the performance of the capacitors, the dielectrics are becoming the main research objectives in academic circle. Ceramics capacitors are difficult to achieve high energy storage density due to their low breakdown field strength, while energy storage density of polymer capacitors is also limited by their low dielectric constant. The ceramic/polymer composites have obtained the improved energy density but their flexibility, stability, and uniformity are poor.^[^ ^1f^ ^]^ In addition to ceramics and ceramic/polymer composites, electrolytic capacitors are also a commonly used type of capacitor. Electrolytic capacitors have higher capacitance and higher volumetric efficiency than film capacitors.^[^ ^2e^ ^]^ Due to these advantages, electrolytic capacitors have been widely used in many fields, such as energy buffers of capacitive circuits, DC bus power balancers of inverters, output voltage regulators.^[^ ^2f^ ^]^ However, electrolytic capacitors are prone to wear and other failures, resulting in short life and safety problems, especially explosions owing to failures in high‐voltage environments. In contrast to the typical dielectric materials of electrolytic capacitors, ceramics, and ceramic/polymer composites, all‐organic polymers demonstrate tremendous potential in dielectric capacitor application because of their natural advantages including mechanical flexibility, low density, ease of processing, low cost, and high operating voltages.^[^ ^2^, ^4^ ^]^ At present, the commercial dielectric capacitors mainly contain film capacitor and multi‐layer ceramic capacitor in industrial circle, of which the most widely used dielectrics are biaxially oriented polypropylenes (BOPP) and the BaTiO_3_ or doped‐BaTiO_3_ ceramics, respectively.^[^ ^5^ ^]^ Taking BOPP as an example, the value of global market exceeds $2 billion, which occupies a large proportion of all available dielectrics. The main reasons for BOPP being employed as the most widely used dielectrics are as follows. BOPP achieves an ultralow dielectric loss of 0.0002–0.0003 at 1 kHz, ultrahigh breakdown strength of 700–750 MV m^−1^, and a long lifetime of >20 kh at a high operating electric field of 200 MV m^−1^ with a high energy efficiency of >95%. In addition, the cost is low, such as the price of a 1mF BOPP capacitor is about $90.^[^ ^5^, ^6^ ^]^ For comparison, some key factors of the commonly used polymer dielectrics are summarized in **Table** **1**.^[^ ^7^ ^]^

**Table 1:** General Characteristics of Typical Commercial Polymer Dielectric Capacitors.^[^ ^7^ ^]^

Dielectric capacitors	Dielectric constant	Glass transition temperature [°C]	Maximum operating temperature [°C]	Voltage breakdown [V µm^−1^]	Dissipation factor %1 kHz	Energy density [J cm^−3^]
Polypropylene (PP)	2.2	<25	85/105 (at 70% of rated voltage)	640	<0.02	1–1.2
Polyester (PET)	3.3	75–80	125	570	<0.5	1–1.5
Polyethylenenaphlate (PEN)	3.2	125	150	550	<0.15	1–1.5
Polyphenylenesulfide (PPS)	3.0	120	125	550	<0.03	1–1.5

As shown in Table 1, the major limitations for BOPP capacitor applications come from two aspects. One of the largest bottleneck of BOPP is the limited energy density due to its low dielectric constant, which will make the applied equipment large and heavy. Another shortcoming is the low operating temperature due to its low glass transition temperature and low melting point, which restricts its application in high‐temperature fields. To tackle these challenges and further improve the comprehensive performance of BOPP‐based capacitors, scientists in the whole world are conducting intensive researches. The goal of going beyond BOPP is achieved by developing polymers with higher dielectric constants, high glass transition temperatures, high breakdown field strengths, and low losses. With the above advantages, the new dielectric polymers can maintain good performance even at high temperature, which is impossible to be obtained in the current BOPP films. Recently, America has started up advanced power electronics and electric motor (APEEM) program^[^ ^5b^ ^]^ in a guideline for future DC‐link capacitors, in which the targets are listed in **Table** **2**.

**Table 2:** The objectives of the advanced power electronics and electric motor program.^[^ ^5b^ ^]^

Performance	Parameter
Capacitance [µF]	>1000
Voltage rating [*V*_DC_]	650–900
Tan*δ* at 10 kHz	<0.005
Equivalent series resistance [mΩ]	<2
Equivalent series inductance [nH]	≤5
Temperature [°C]	140
Ripple current [*A*_rms_]	100
Failure mode	benign
Lifetime [h]	>20 000
Volume [L]	≤0.6
Cost [$]	≤30

In the past decade, the polymer‐based ceramic dielectric composites have been widely investigated because the tremendous energy density can be obtained. For example, the highest *U*_d_ even was as high as 40 J cm^−3^, which was more than 30 times larger than that of BOPP. However, it is far from enough to replace the commercial BOPP. Therefore, a comprehensive comparison between polymer dielectrics in laboratory and industries are conducted. As shown in **Table** **3**, there is a large difference in ways of materials, preparation method, dielectric constant, energy density, insulation, mechanical properties, machinability, and cost.^[^ ^6^, ^8^ ^]^ Considering the demands for industrial applications, lots of issues should be addressed for polymer‐based ceramic dielectric composites, especially in the aspects of machinability, stability, and cost.

**Table 3:** The comparison between polymer dielectrics in laboratory and industrial.^[^ ^6^, ^8^ ^]^

	In laboratory	In industry
Materials	PVDF‐based ceramic composites, PI, poly(methyl methacrylate) PMMA, PP‐based composites	BOPP, BOPET
Preparation method	Solution method	Melt extrusion
Dielectric constant	High	Low
Energy density	High (≈40 J cm^−3^)	Low (3–5 J cm^−3^)
Insulation	Decreased breakdown strength and increased loss	Stable
Mechanical properties	Uneven film flexibility and mechanical strength	Stable mechanical properties
Machinability	Continuous large area processing is not possible	Continuous and large area processing
Cost	High	Low

**Figure 3:**
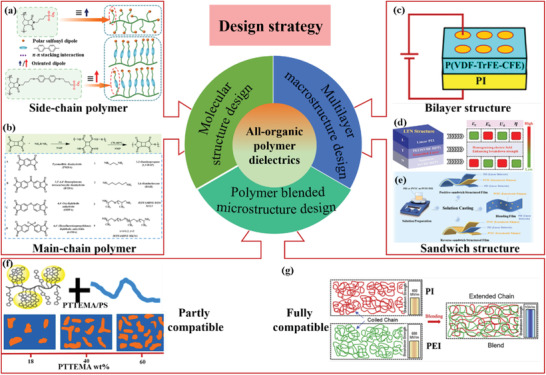
Strategies for polymer dielectrics with improved performance. a) Reproduced with permission.^[^ ^14e^ ^]^ Copyright 2021, American Chemical Society. b) Reproduced with permission.^[^ ^14d^ ^]^ Copyright 2014, American Chemical Society. c) Reproduced with permission.^[^ ^14c^ ^]^ Copyright 2020, American Chemical Society. d) Reproduced with permission.^[^ ^14b^ ^]^ Copyright 2021, Wiley‐VCH. e) Reproduced with permission.^[^ ^14a^ ^]^ Copyright 2021, Royal Society Chemistry. f) Reproduced with permission.^[^ ^14f^ ^]^ Copyright 2015, American Chemical Society. g) Reproduced with permission.^[^ ^14g^ ^]^ Copyright 2021, Elsevier.

Nowadays, a wide variety of key topics on polymer‐based ceramic composites dielectrics have been reviewed, such as nanocomposites for high *U*_d_ capacitor applications,^[^ ^9^ ^]^ methods of interface design by introducing the range of interface layers and structures,^[^ ^10^ ^]^ high‐k dielectric nanocomposites,^[^ ^11^ ^]^ high‐temperature dielectric nanocomposites,^[^ ^12^ ^]^ and multilayered dielectric nanocomposites.^[^ ^1^, ^13^ ^]^ In this review, we aim to explore the recent progress on approaches to developing all‐organic polymer dielectrics with predominant performance, including increased relative permittivity, suppressed dielectric loss, enhanced breakdown strength and *U*_d_, improved film‐formation and processability, and stability in high temperature. According to literature reports, all‐organic polymer dielectrics can be divided into three categories including molecular structure design, polymer blends, and multilayered structure, as illustrated in **Figure** **3**. These strategies will be overviewed in detail next in the aspects of side‐chain dipolar polymer and main‐chain dipolar polymer based on molecular structure design, partly compatible blended dielectric polymers and fully compatible blended dielectric polymers, bilayer structure, and sandwich structure dielectric polymers. Meanwhile, the state‐of‐the‐art dielectric constant, breakdown strength, *U*_d_, and efficiency of typical polymer dielectrics with the strategies of molecular design, polymer blends, and layers structured polymer are shown in Figure 1b,c, indicating that every kind of dielectric polymers exhibits the superior dielectric properties.

## 2 Architectures for Polymer Dielectrics

### 2.1 Molecular Structure Design

**Figure 4:**
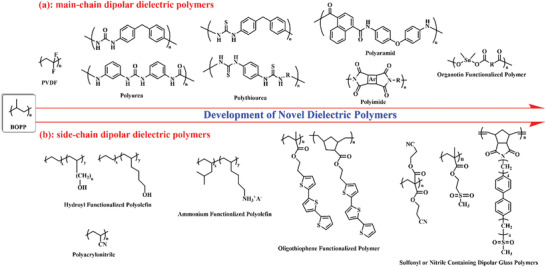
A summary of selective main‐chain dipolar dielectric polymers and side‐chain dipolar dielectric polymers developed in recent years.

Molecular structure design is a feasible method to control the intrinsic properties of polymer dielectric materials because not only the different functional groups can be simultaneously linked to the constitutional unit of polymer and but also the condensed state structure of polymer can be tailored, including crystalline, liquid crystalline and amorphous state. For the dielectric polymers, the molecular structure design is focused on dipolar polymers.^[^ ^2^, ^16^ ^]^ A polar group refers to a group whose positive and negative charge centers do not overlap and the polarity comes from the different electronegativity between the elements of a bond. The elements with strong electronegativity can withdraw the electron cloud from the adjacent element, resulting in that the former presents the partial negative charge and the latter shows the partial positive charge. The polarity can be characterized by a dipole moment. The dipole moment is larger, the polarity of the group is greater, resulting in the intense orientation polarization. Compared with non‐polar and weakly polar materials (linear dielectrics without permanent dipoles), such as polyethylene (PE), PP, polystyrene (PS), PET, polar polymers (nonlinear dielectrics) have a higher *Ɛ*_r_ because the dipoles usually do not cancel out each other causing the reinforcement of individual dipole moments. It should be noted that the polar nature of the polymers is collectively decided by the presence of polar groups and chain geometry. For example, polytetrafluoroethylene is a kind of nonpolar polymer associated with the symmetry of molecular structural unit. Based on the different position of polar groups attached to either main or side chains of the polymer, the dipolar dielectric polymers are classified into two kinds: main‐chain dipolar dielectric polymers and side‐chain dipolar dielectric polymers. As can be seen in **Figure** **4**, when the common dipolar group, such as urea group (—NH—CO—NH—), thiourea group (—NH—CS—NH—), imide group (—N(—CO)_2_—), sulfone group (R—SO_2_—R′), amide group (R—CO—NH—), hydroxyl group (—OH), sulfonyl group (—SO_2_CH_3_), cyano group (—C≡N), and so on, is introduced into the main‐chain, dipolar polymer is named the main‐chain dipolar dielectric polymers. When dipolar group is added into the side‐chain, the dipolar polymer is called side‐chain dipolar dielectric polymers. These two kinds of dielectrics will be discussed in detail in the following sections.

#### 2.1.1 Main‐Chain Dipolar Dielectric Polymers

In this section, dielectric polymers consisting of dipolar groups in main chain will be overviewed. For main‐chain dipolar dielectric polymers, the dipolar groups like urea (4.56 D), thiourea (4.89 D), imides, urethane are usually selected to attach to the main‐chain of polymers.^[^ ^15a^ ^]^ Through changing the chemical structures related to the rigid, semi‐flexible, or flexible polymer chain and the interaction between polymer chains (Van der Waals' force and hydrogen‐bond interaction), the mechanical, film‐forming, thermostable, dielectric, and energy storage properties of main‐chain dipolar dielectric polymers can be regulated in order to meet the practical application.^[^ ^15a,b^ ^]^ For example, a flexible segment was added into the main chain to increase the movement of the dipole and the dipole polarization at the molecular level. Based on this consideration, the m‐PhPU (formed by condensation polymerization of m‐phenylenediamine and diphenyl carbonate) and the MePTU (formed by condensation polymerization of thiourea and formaldehyde) were synthesized by Yash et al.,^[^ ^15a^ ^]^ of which the *Ɛ*_r_ reached 5.7. At the same time, a *U*_d_ of 13 J cm^−3^ and an efficiency of 90% under a high electric field of 670 MV m^−1^ were achieved in the m‐PhPU. The *Ɛ*_r_ of PDTC‐HK511(form para‐phenylene diisothiocyanate and Jeffamine HK511 polymerization), TDI‐EDR148(form toluene‐2,4‐diisocyanate and JeffamineEDR148 polymerization) and BTDA‐HK25 (form 3,3′,4,4′‐benzophenone tetracarboxylic dianhydride, Jeffamine HK511, and hexane‐1,6‐diamine polymerization) fabricated by Li et al.^[^ ^15b^ ^]^ were 5.5, 6.4, and 4.5, respectively. The corresponding maximum energy densities were 11 J cm^−3^, 13 J cm^−3^, and 12 J cm^−3^, respectively. The efficiencies of three polymers were all higher than 90%, which were much higher than those of untreated polymers.

The dipolar glassy polymer is a polymer with a high glass transition temperature (*T*_g_) and significant dipolar polarization at the sub‐glass transition temperature. The high *T*_g_ can prevent the loss of electronic and ionic conduction. At the same time, the larger temperature difference between the *T*_g_ and sub‐*T*_g_ (e.g., *γ* and *β* relaxations) caused by the rotation of the dipoles is more suitable for high *U*_d_ and low loss is suitable for high‐temperature applications.^[^ ^11c^ ^]^ Xu et al.^[^ ^17^ ^]^ have designed and prepared a new type of main‐chain dipolar glassy polymer in which an ether ketone structure and a polyaramid (PA) naphthalene ring were imbedded into the main chain to decrease the density of amide bonds in the main chain and reduce the strength of hydrogen bonds between the polymer chains. Due to the increase in the mobility of the dipole groups, the *Ɛ*_r_ of this polymer was increased to 4.5, and the dielectric loss remained at 1%. In addition, the polymer possessed a high *T*_g_ of 251 °C and good solubility. The glassy polymer with special molecular structure exhibited excellent energy storage performance at elevated temperature, for example, the *U*_d_ and energy efficiency reached 2.1 J cm^−3^ and 86.8% at the temperature of 200 °C. Amorphous aromatic nylon (Selar), known as copolymer 6I/T, is an amorphous copolymer composed of hexamethyl ethylenediamine, isophthalic acid, and terephthalic acid.^[^ ^18^ ^]^ Zhang et al. have investigated the origin of ferroelectricity for Selar which belonged to the commercial main‐chain dipolar glass polymers.^[^ ^19^ ^]^ The experiment results showed that the primary contribution to ferroelectric switching came from cooperative segmental motions in the main‐chain of dipolar glass nylon. Consequently, it was considered that semicrystalline aromatic nylons with extended thermal annealing could suppress ferroelectric switching and present the high efficiency.

Polyimides (PIs) display the high *T*_g_ and excellent mechanical properties, and are widely used in high temperature or highly corrosive environments.^[^ ^20^ ^]^ Treufeld et al.^[^ ^21^ ^]^ thought that by introducing high‐polarity nitrile groups into the main‐chain of PI, the *Ɛ*_r_ of this polymer would be increased. As a result, a series of new PIs by adding CN dipoles to the *π* structure were synthesized, of which the ∆*ε*_r_^′^ (the difference between the real part of relative permittivity at 190 °C, 1 kHz and that at 150 °C, 1 kHz.) was increased from 0.05 to 0.76. However, as the *Ɛ*_r_ increased, the dielectric loss of the polymer was also increased in this work. **Table** **4** summarizes the properties of some main‐chain dipolar dielectric polymers.

**Table 4:** Energy storage properties of main‐chain dipolar dielectric polymers

Polymer	Dipolar groups	Dielectric constant	Breakdown strength *E*_b_ [MV m^−1^]	Energy storage density [J cm^−3^]	Charge‐discharge efficiency	References
M‐PhPU	Urea	5.7	670	13	>90％	^[^ ^15a^ ^]^
MePTU	Thiourea	5.7	‐	‐	>90％	^[^ ^15a^ ^]^
PDTC‐HK511	Thiourea	5.5	780	11	>90％	^[^ ^15b^ ^]^
TDI‐EDR148	Thiourea	6.4	780	13	>90％	^[^ ^15b^ ^]^
BTDA‐HK25	Imide	4.5	780	12	>90％	^[^ ^15b^ ^]^
PDTC‐ODA	Thiourea	4.52	704	>8	>70%	^[^ ^15c^ ^]^
PDTC‐MDA	Thiourea	4.08	714	>9.5	>70%	^[^ ^15c^ ^]^
PDTC‐PhDA	Thiourea	4.89	‐	‐	‐	^[^ ^15c^ ^]^
PDTC‐HDA	Thiourea	3.67	685	9.3	>70%	^[^ ^15c^ ^]^
PDTC‐HK511	Thiourea	6.09	602	>6	>85%	^[^ ^15c^ ^]^
Thiophosgene‐MDA	Thiourea	3.84	677	>9	>70%	^[^ ^15c^ ^]^
Meta‐PU	Urea	5.6	670	13	91%	^[^ ^22^ ^]^
Meta‐PTU	Thiourea	6	500	7.5	‐	^[^ ^23^ ^]^
Aliphatic polythiourea	Thiourea	5.5	770	17	‐	^[^ ^23^ ^]^
PEKNA	Amide	4.5(200 °C)	>350(200 °C)	2.1(200 °C)	86.8％(200 °C)	^[^ ^17^ ^]^
Polyimide B4	Imide	7.8	676	15	‐	^[^ ^14d^ ^]^
P(MDA/MDI)	Urea	4.2	800 500(180 °C)	12 6(180 °C)	>90%	^[^ ^15d^ ^]^
ArPTU	Thiourea	‐	1100	24	‐	^[^ ^24^ ^]^
ArPU	Urea	4.2	800	13	‐	^[^ ^23^ ^]^
PEEK‐SO_2_	Sulfonyl	5	300	2.35	>85%	^[^ ^25^ ^]^

In addition to the polythiourea (PTU), polyureas (SPUA), polyurethanes(PU), and PIs, polyvinylidene fluoride (PVDF) and PVDF‐based copolymers are another kind of main‐chain dipolar dielectric polymers and considered to have great potential in energy storage because of their strong dipole moment of C—F bonds and spontaneous polarization of crystalline phase. PVDF presents at least four phases. Due to the existence of polar *β* phase, the PVDF shows ferroelectricity. However, the energy density and energy efficiency of PVDF are limited, which is associated with the large residual polarization and relatively low saturation electric field. To address the various issues confronted in the application of PVDF as dielectric materials, two approaches are carried out at present including chemical method and physical method. By introducing physical, chemical defects or polymers into the main chain of PVDF, the PVDF can be converted from ordinary ferroelectrics into ferroelectric relaxors, resulting in reduced residual polarization and increased saturation electric field.^[^ ^26^ ^]^ For instance, comonomers of chlorotrifluoroethylene (CTFE) or hexafluoropropene (HFP) are introduced into the main chain of PVDF to increase the interface area and reduce the paraelectric phase, leading to higher *Ɛ*_r_ and *U*_d_ than that of pure PVDF.^[^ ^27^ ^]^ On the other hand, the *β* phase of PVDF‐based polymers can be enhanced by uniaxial stretching, heat treatment, electrodeposition, and other methods.^[^ ^28^ ^]^

**Figure 5:**
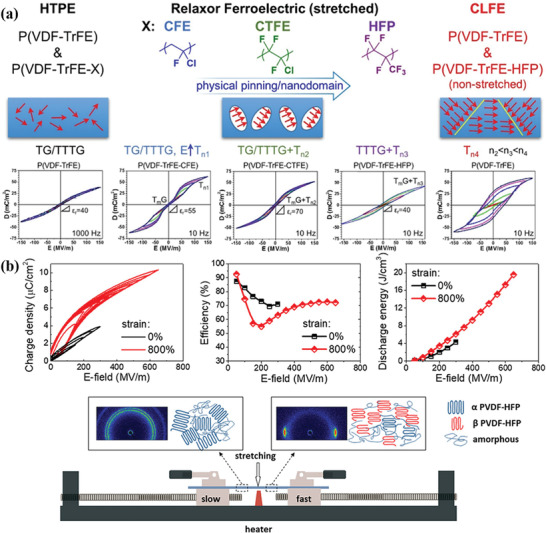
a) Structure–property relationship between HTPE P(VDF‐TrFE)/terpolymers, stretched RFE terpolymers (X = CFE, CTFE, and HFP), and CLFE P(VDF‐TrFE)/unstretched P(VDF‐TrFE‐HFP). Reproduced with permission.^[^ ^29^ ^]^ Copyright 2017, American Chemical Society. b) Comparison of energy storage performance of as‐prepared samples and morphology development upon stretching for ferroelectric polymers and a laboratory zone‐stretching machine. Reproduced with permission.^[^ ^36^ ^]^ Copyright 2020, American Chemical Society.

Chemical pinning can hinder the synergistic effect of crystal dipoles to form stable nanodomains. The comonomer HFP with large volume is generally excluded from the crystal structure, and strong pinning effect cannot be built. Li et al.^[^ ^29^ ^]^ have synthesized P(VDF‐TrFE‐HFP) via a suspension ternary copolymerization method, and then a mechanical stretching method was used to pull the HFP originally outside the crystal region into the crystal structure (see **Figure** **5**a). The dipole physical pinning effect of VDF/TrFE was produced, and nano‐ferroelectric domains with strong physical pinning effect were established. This is the direct reason why the relaxor ferroelectric phenomenon can be observed on the single drawn stretched film.

However, the presence of residual metal ions will cause metal pollution and will seriously deteriorate the dielectric properties of the material. To solve this problem, Tan et al.^[^ ^32^ ^]^ have proposed a method to graft PMMA onto P(VDF‐CTFE) by light‐induced metal‐free ATRP using C—Cl bond as raw material. Without the influence of metal ions, the polymer showed lower dielectric loss and improved breakdown strength. It is difficult for linear polymers to maintain good dielectric properties at high temperatures, but the cross‐linked molecular structures can inhibit the transport of carriers in the polymer even at high temperatures. Li et al.^[^ ^33^ ^]^ have prepared an all‐organic polymer cross‐linked P(VDF‐CTFE) with excellent performance at high temperature. Since the cross‐linking molecule can effectively limit the charge transportation and reduce the conduction loss under high temperature and high external electric field, the polymer exhibited excellent dielectric properties at high temperature.

Employing physical method, such as stretching is a good way to improve the *U*_d_ and energy efficiency of the dielectrics.^[^ ^13^, ^27^, ^34^ ^]^ Uniaxial/biaxial stretching is a process that can reduce the film thickness and improve the quality of the film for the manufacture of capacitor films. During the stretching process, the phase, crystal orientation, interface property, and crystallite size of the polymer will be regulated, which will enormously affect the dielectric properties and energy storage performance of the dielectrics.^[^ ^35^ ^]^ For instance, the changes in structure and dielectric properties of the P(VDF‐HFP) (see Figure 5b) under different stretching ratios were studied by Yuan et al.^[^ ^36^ ^]^ With the increase of strain, most of *α* phase will be evolved into *β* phase and the grain size will be decreased. Therefore, the dipole moment gradually varied to the parallel electric field. This made the polymer obtain high *Ɛ*_r_ and breakdown strength when the tensile ratio was 800%, and an *U*_d_ of 20 J cm^−3^ was attained.

In order to obtain materials with better dielectric and ferroelectric properties, Meng et al.^[^ ^37^ ^]^ have researched the energy storage performance of various PVDF‐based polymer films synthesized by using melt extrusion and hot‐pressing methods. The results showed that the polymer chains in the amorphous region will be arranged along the extrusion direction in the film after melt extrusion, which is more suitable for applying a vertical electric field to the film than the randomly arranged polymer. In addition, the arrangement of polymer chains in the amorphous region along the extrusion direction will also expand the interface region, leading to the increased *Ɛ*_r_. Because the shear force acts directly on the flowing melt, more defects will be produced in melt extrusion, which makes the domain wall density of the film produced by melt extrusion higher than that of the films by hot pressed, resulting in higher *Ɛ*_r_ at low frequency. At high frequencies, the *Ɛ*_r_ of the extruded film was lower than that of the hot‐pressed film due to the longer relaxation time of the domain wall.

As mentioned above, heat treatment is a kind of strategy to promote the performance of polymer dielectrics, referring to the annealing temperature and annealing time. Different annealing temperature and annealing time play a great influence on the crystal morphology, grain size, and dielectric properties of PDVF‐based polymers. The rapid differential scanning calorimetry was performed to control the annealing time and annealing temperature of P(VDF‐CTFE) by Chen et al.^[^ ^38^ ^]^ The authors found that P(VDF‐CTFE) annealed at low temperature had lower crystallinity than that of P(VDF‐CTFE) annealed at high temperature, causing smaller crystal domains and a higher dipole moment response. However, P(VDF‐CTFE) presented higher remanent polarization after low‐temperature annealing. The ferroelectric phase formed after low‐temperature annealing was more than that by high‐temperature annealing. Hence, the discharge efficiency of the polymer decreased after annealing at low‐temperature, but the higher *Ɛ*_r_ can be obtained. In the process of rapid cooling, smaller domains and lower crystallinity will be observed, and the thickness of paraelectric crystal will be reduced. Therefore, with the increase in cooling rate, the electrical saturation polarization, *Ɛ_r_*, *U*_d_, and dielectric loss of polymer will increase. A similar conclusion was reported by Xia et al.,^[^ ^39^ ^]^ who have investigated the effect of annealing temperature on the crystallinity and energy storage properties of the P(VDF‐*co*‐CTFE). The *Ɛ*_r_ and dielectric loss of P(VDF‐co‐CTFE) after low‐temperature treatment were increased slightly at low frequency, but of which were decreased at high frequency because of the effect of amorphous dipole. After annealing at low‐temperature, the crystallinity and grain size of P(VDF‐co‐CTFE) were decreased, and breakdown strength, maximum polarization, and residual polarization of the polymer improved. The results revealed that under the same conditions of electric field and frequency, lower annealing temperature was conducive to the improvement of the *Ɛ*_r_ and *U*_d_ of the polymers.^[^ ^38^, ^39^ ^]^ Divya et al.^[^ ^40^ ^]^ have studied the effects of heat treatment and polarization on the dielectric properties of the P(VDF‐HFP) film which was fabricated via spin coating technology. During the melt‐quenching process, the Ɛ_r_ of the polymer increased significantly. The reason was that electric dipoles generated by F^−^ and H^+^ were arranged along the direction of the applied electric field to form an all‐trans plane zigzag configuration. This phenomenon makes the *β* phase of the poled polymer increase, and the polarization force and coercivity of the polymer increase significantly, which further increases the dielectric constant and improves the energy storage performance of the dielectrics.

#### 2.1.2 Side‐Chain Dipolar Dielectric Polymers

When the dipolar groups like ammonium‐functionalized, hydroxyl, thiophene, sulfonyl, and cyano are linked to the side chain of the polymers, the dipole is easier to rotate than that of main‐chain, resulting in fantastic dielectric properties.^[^ ^21^, ^41^ ^]^ Yauhen Sheima et al.^[^ ^42^ ^]^ have investigated the effect of the type, strength, and volume of the polar side chain groups on the *T*_g_ and *Ɛ*_r_ of polysiloxanes. The results showed that the *T*_g_ increased with the polar group content and the strength of the polar group. A similar trend was observed for the *Ɛ*_r_ when the *T*_g_ of the polymer was below 0 °C. Smaller polar groups tend to obtain a smaller improvement of *T*_g_, and an increased linker length helps to decrease *T*_g_, which is generally favorable for the improvement of *Ɛ*_r_. Although the authors found that the highest *Ɛ*_r_ of 27.7 with a *T*_g_ below room temperature was obtained in the design polysiloxanes, the high dielectric loss was also achieved originating from the high polarization loss and conductive loss.

In order to get high *Ɛ*_r_, the flexible polymer chain is a good choice for side‐chain dipolar dielectric polymers. However, the movement of polymer chain will contribute to supernal conductive loss and polarization loss originating from drastic molecular collisions under an applied electric field. Consequently, the side‐chain dipolar dielectric polymers should have a high *T*_g_ to decrease the dielectric loss, such as rigid or semi‐rigid polymer chain, including main‐chain of PS, polymethacrylate, polynorbornene, aromatic, cycloaliphatic, heterocyclic. Studies have fully proved that the close packing of polymer chains can increase the breakdown field strength. In particular, polyacrylonitrile (iso‐PAN) is easier to form dense packing than atactic polyacrylonitrile (ata‐PAN).^[^ ^43^ ^]^ Yu Wang et al.^[^ ^44^ ^]^ have prepared the isotactic iso‐PAN via template polymerization using the 2D layered space of CoCl_2_ crystal as a template. Due to the large dipole moment (4.3 D) of nitrile group and high stereoregularity, iso‐PAN presented a high *U*_d_ of 6.7 J cm^−3^ at 660 MV m^−1^, nearly six times that of atactic polyacrylonitrile and BOPP. In addition, the charge–discharge efficiency reached 90%.

In order to improve the stability of the polymer and maintain a higher energy storage density and efficiency, some other attempts have been proposed. Poly(acrylonitrile butadiene styrene) (ABS) possesses a high *T*_g_, which is beneficial to maintain the stability under high temperature. With the help of polar group cyano, ABS produces a strong polarization effect. Moreover, the ABS consists of linear polymer PS, which is beneficial to the high charge and discharge efficiency. In this case, the ABS thin film prepared via solution casting method by Wen et al.^[^ ^45^ ^]^ showed great energy storage performance and temperature stability. For example, under an electric field of 525 MV m^−1^ and at room temperature, an *U*_d_ of 7.3 J cm^−3^ and a charge–discharge efficiency of 80% were discovered. More importantly, the ABS polymer film could still hold an U_d_ of 6.7 J cm^−3^ and a charge–discharge efficiency of 75% at a high temperature of 120 °C. The cyclic test revealed that the ABS film maintained the stable *U*_d_ and charge–discharge efficiency under 10^5^ cycles.

Through using LiAlH_4_ as a reducing agent to reduce the MMA group content in P(St‐MMA), Liu et al.^[^ ^15e^ ^]^ have synthesized a terpolymer poly(styrene‐methyl methacrylate‐methallyl alcohol) (P(St‐MMA‐MAA)). The introduction of ‐OH can increase the *Ɛ*_r_ and breakdown strength of the copolymers. As a result, a high *U*_d_ of 14.5 J cm^−3^ and efficiency of 93% were obtained at 110 °C. This work provided a new strategy to alleviate the long‐term contradiction between high energy storage density and low energy loss. According to the reports,^[^ ^46^ ^]^ introduction of PS or poly(ethyl methacrylate) (PEMA) on the copolymer would result in the formation of an insulating layer around the polar crystal domains, leading to the increase of the breakdown field strength and the decrease of the dielectric loss. For instance, Guan et al.^[^ ^46b^ ^]^ have prepared the P(VDF‐TrFE‐CTFE)‐g‐PS by grafting low polarizability PS. And PS as side‐chain forms the insulating layer on the outer layer of P(VDF‐TrFE) due to its excellent insulating properties, which can effectively reduce the overall loss of the polymer. Compared with pure P(VDF‐TrFE), the loss of P(VDF‐TrFE‐CTFE)‐g‐PS have been significantly decreased. In addition, Li et al. have synthesized P(VDF‐TrFE‐CTFE)‐g‐PEMA by an ARGET‐ATRP process. Compared with the P(VDF‐TrFE) random copolymer, PEMA can induce domain confinement and polarization confinement in copolymer. Therefore, the breakdown field strength of the composite can be increased and the loss can be reduced at the same time.

**Figure 6:**
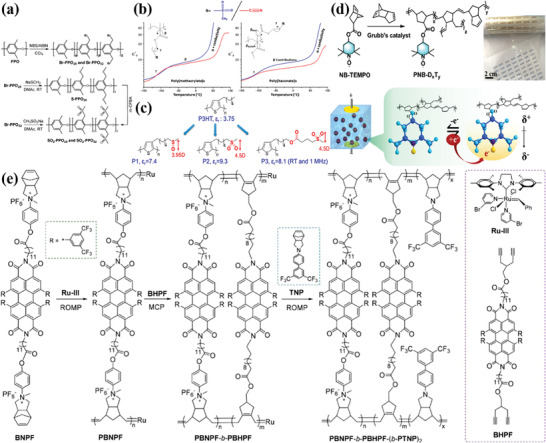
a) Schematics of the synthetic routes for the sulfonylated PPOs SO_2_‐PPO_25_ and SO_2_‐PPO_52_. Reproduced with permission.^[^ ^49^ ^]^ Copyright 2018, Wiley‐VCH. b) Schematic diagram of the dielectric temperature spectrum and structure of poly(methacrylate)s and poly(itaconate)s. Reproduced with permission.^[^ ^51^ ^]^ Copyright 2018, American Chemical Society. c) Schematic of the structure of P3HT, P1, P2, and P3. Reproduced with permission.^[^ ^52^ ^]^ Copyright 2018, American Chemical Society. d) Schematics of the synthetic routes for PNB‐DxTy and single radical capturing electrons under an electric field and the resulting dipole. Reproduced with permission.^[^ ^15h^ ^]^ Copyright 2020, American Chemical Society. e) Synthetic scheme of double (double‐stranded) block copolymer. Reproduced with permission.^[^ ^15g^ ^]^ Copyright 2018, American Chemical Society.

Considering the potential applications, side‐chain dipolar glassy polymer proposed by Zhu et al. is a kind of ideal polymer dielectric due to the advantages of high *U*_d_, high‐temperature stability, and low loss.^[^ ^11c^ ^]^ For the side‐chain dipolar glassy polymer, high *T*_g_ and low sub‐*T*_g_ transitions (e.g., *β* and *γ* relaxations) of dipolar entities should be paid attention to. The high glass transition temperature can effectively restrict the loss of electron and ion conduction, thereby increasing the charge–discharge efficiency. The low sub‐*T*_g_ transitions of dipolar group can increase *Ɛ*_r_. In order to make the temperature difference between the *T*_g_ and low sub‐*T*_g_ large enough, on the one hand, the content of polar groups should be low, on the other hand, the size of dipolar groups should be less than 0.6 nm.^[^ ^47^ ^]^ The combination of these two points ensures that the *T*_g_ is high and the permanent dipole can rotate in the side‐chain dipolar glassy polymer.^[^ ^47^, ^48^ ^]^ For example, Zhang et al.^[^ ^49^ ^]^ have reported a kind of new high‐temperature dipolar glass polymer through post‐polymer functionalization, poly(2,6‐dimethyl‐1,4‐phenylene oxide)(SO_2_‐PPO) (see **Figure** **6**a). Attributing to the small size and large dipole moment (4.25 D) of the methylsulfonyl groups, high *Ɛ*_r_ of 5.9 and 8.2 were obtained in SO_2_‐PPO_25_ and SO_2_‐PPO_52_ at 1 kHz and room temperature, respectively. Additionally, the rigidity of PPO main chain contributed to a high *T*_g_, a high breakdown strength, and a low dielectric loss. Ultimately, the *U*_d_ of SO_2_‐PPO_25_ reached 22 J cm^−3^ with a low dissipation factor of 0.003 at high temperature of 150 °C. Wei et al.^[^ ^50^ ^]^ synthesized a dipolar glass polymer containing polar sulfone side group, named poly(2‐(methylsulfonyl)‐ethyl methacrylate) (PMSEMA). The *T*_g_ of this polymer was 109 °C. The *Ɛ*_r_ was significantly improved to 11.4 at a frequency of 1 Hz and 10.5 at a frequency of 1 kHz. Novel polyitaconates with sulfone or nitrile side group were fabricated by Bonardd et al.,^[^ ^51^ ^]^ as illustrated in Figure 6b. The testing results indicated that all polymers produced high *Ɛ*_r_ values between 7 and 10 at 25 °C and 1 kHz and obtained a relatively low dielectric loss values of ≈0.02. Although the conjugated polymer has high dipole moment, poly(3‐alkylthiophene) possesses low *Ɛ*_r_ because the *π*‐conjugated dipole cannot be freely rotated under the alternating electric field. Consequently, Wang et al.^[^ ^52^ ^]^ synthesized functionalized poly(3‐alkylthiophene)s (P3AT) with highly dipolar methylsulfinyl or methylsulfonyl end groups in the alkyl side chains. On account of efficient rotation of highly polar methylsulfinyl and methylsulfonyl side groups, the *Ɛ*_r_ of three conjugated polymers (P1, P2, P3) increased from 3.75 of the original polymer to 7.4, 9.3, and 8.1, respectively, as shown in Figure 6c.

In contrast to linear polymers with dipolar side‐chain, the crosslinked polymers with dipolar side‐chain can effectively improve the film‐forming property, the breakdown strength, energy storage density, thermal stability, and charge–discharge efficiency of the polymers.^[^ ^14^, ^53^ ^]^ Via continuously adjusting the molar ratio between ring‐like alicyclic amine (ACA) and linear polyether amine (PEA) groups, a superhigh energy storage density of 9.12 J cm^−3^ under an electric field of 550 MV m^−1^, and an efficiency of 90% were achieved. Li et al.^[^ ^15h^ ^]^ have prepared a series of flexible poly(dicyclopentadiene norbornene‐ended 4‐hydroxy‐2, 2, 6, 6‐tetramethylpiperidin‐*N*‐oxy) (PNB‐DxTy) copolymers, as shown in Figure 6d. In the copolymers, a cross‐linked network was built by dicyclopentadiene groups and the 4‐hydroxy‐2,2,6,6‐tetramethylpiperidin‐*N*‐oxy (TEMPO) groups were utilized to provide a stable radical to capture electrons under the action of an electric field. The results showed that the (PNB‐D500T100) copolymer presented *U*_d_ of 10.6 J cm^−3^ together with promising charge–discharge efficiency of 92% at 500 MV m^−1^, and good stability. Azobenzene is a material with high dipole moment and polarizability because of its strong push‐pull substituents. On the other side, it can undergo anti‐cis‐trans isomerization under certain light or heating conditions.^[^ ^54^ ^]^ Based on these properties, co‐crosslink polymers with azobenzenes side‐chain were fabricated via a simple one‐step process by Zhang et al.^[^ ^55^ ^]^ As expected, high *Ɛ*_r_ and high breakdown strength were obtained in the designed elastomers.

Block copolymers can separate on a molecular level (10–100 nm) because of the covalent link between the blocks as well as their immiscibility, showing different well‐ordered morphologies and fascinating dielectric properties. For example, Zhu et al.^[^ ^56^ ^]^ have synthesized block copolymer with push‐pull azobenzene pendants using ring‐opening metathesis polymerization method. This block copolymer can self‐assemble into a core‐shell nanostructure with high dipole and interface polarization. Combined with the high dipole moment with unique nanostructure, PFANP_200_‐b‐PNANP_100_ possessed a *Ɛ*_r_ as high as 19.1 and maintain a dielectric loss of 0.018 at 1 kHz. Under an electric field of 240 MV m^−1^, the *U*_d_ of PFANP_200_‐b‐PNANP_100_ reached 5.54 J cm^−3^ with an efficiency of 82.1%. Chen et al.^[^ ^15g^ ^]^ have prepared ionic‐conjugated hybrid conductive segments‐contained bis(double‐stranded) conductive‐insulating block copolymer PBNPF‐b‐PBHPF‐(b‐PTNP)_2_ (see Figure 6e). The block copolymer PBNPF‐b‐PBHPF‐(b‐PTNP)_2_ with the regular hollow nanosphere morphology displayed a high *Ɛ*_r_ value of 30 owing to the strong dipolar, electronic, and interfacial polarizations. In addition, the polymer achieved a dielectric loss of 0.02, an energy density of 8.33 J cm^−3^ with a high charge–discharge efficiency of 83.7%, which were superior to those of the same copolymers with irregular nanostructure. (**Table** **5**)

**Table 5:** Energy storage properties of side‐chain dipolar dielectric polymers

Polymer	Dipolar group	Dielectric constant	Breakdown strength [MV m^−1^]	Energy storage density [J cm^−3^]	Efficiency	Ref.
Iso‐PAN	Nitrile	‐	660	6.7	90％	^[^ ^44^ ^]^
ABS	Cyano	‐	525; 400(120 °C)	7.3; 6.7(120 °C)	80％; 75％(120 °C)	^[^ ^45^ ^]^
PMSEMA	Sulfone	11.4	283	4.54	‐	^[^ ^50^ ^]^
PP‐OH‐3	Oxhydryl	‐	600	7.42	‐	^[^ ^57^ ^]^
P(St‐MMA‐MAA)	Hydroxyl	4.2	710	14.5	93％	^[^ ^15e^ ^]^
PMP‐(NH_3_)_3_PO_4_^3−^‐4.5	Amino	>5	612	7.4	93％	^[^ ^15f^ ^]^
SO_2_‐PPO_25_	Methylsulfonyl	5.9	‐	22	91％	^[^ ^49^ ^]^
SO_2_‐PPO_52_	Methylsulfonyl	8.2	‐	24	70％	^[^ ^49^ ^]^
ACA‐PEA	Cyano	‐	‐	9.12	90％	^[^ ^16b^ ^]^
PFANP_200_‐b‐PNANP_100_	Azobenzene; nitro	19.1	240	5.54	82.1％	^[^ ^56^ ^]^
PBNPF‐b‐PBHPF‐(b‐PTNP)_2_	Imide	30	370	9.95	85.7％	^[^ ^15g^ ^]^
PNB‐D_500_T_100_	TEMPO	≈3	500	10.6	92％	^[^ ^15h^ ^]^
Poly[3‐(6‐methylsulfinylhexyl)thiophene]	Methylsulfinyl	7.4	‐	‐	‐	^[^ ^52^ ^]^
Poly[3‐(6‐methylsulfonylhexyl)thiophene]	Methylsulfonyl	9.3	‐	‐	‐	^[^ ^52^ ^]^
Poly[3‐(6‐(3‐methylsulfonylpropylcarbonyloxy)hexyl)thiophene]	Methylsulfonyl	8.1	‐	‐	‐	^[^ ^52^ ^]^

### 2.2 Polymer/Polymer Blended Dielectrics

In general, the synthetic process of dielectric polymers based on the method of molecular structure design is complex, which is difficult to realize large‐scale production. The blend is an economical strategy to prepare high‐performance polymer dielectrics.^[^ ^58^ ^]^ Theoretically, blended polymer can combine the advantages of two different polymers to improve the energy storage performance of the material via tailoring the condensed structure of polymer matrix and the function of organic polymer fillers. Therefore, polymer blend dielectrics will be discussed in depth in this section.

**Figure 7:**
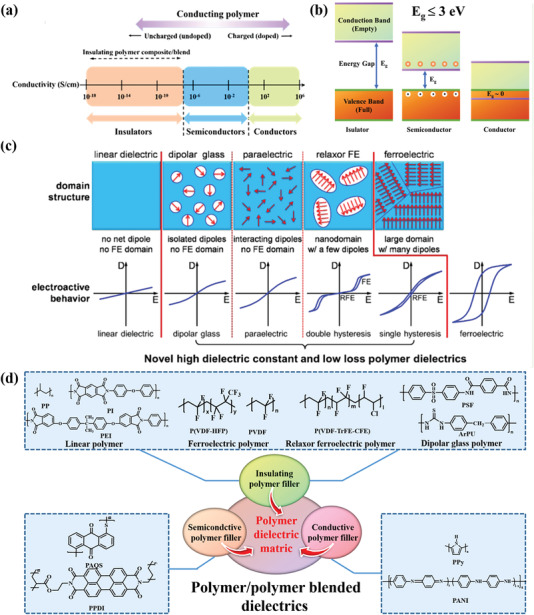
The polymer types based on a) conductivity and b) band gap. Reproduced with permission.^[^ ^1b^ ^]^ Copyright 2020, Wiley‐V C H. c) The kinds of dielectric polymers according to the P‐E loops. Reproduced with permission.^[^ ^13a^ ^]^ Copyright 2017, American Chemical Society. d) The varieties of polymer/polymer blended dielectrics.

Based on the difference of electric conductivity or bandgap (see **Figure** **7**a,b), the polymers can be divided into three kinds, including insulating polymers, semiconductive polymers, and conductive polymers. A satisfactory dielectric polymer should have high flexibility, high temperature stability, high breakdown strength, low dielectric loss, and a wide bandgap in its energy band diagram, as shown in Figure 7b. Compared with insulating polymers, semiconductive polymers with lower bandgap (≤3 eV) will obtain high electronic polarization, resulting in high *Ɛ*_r_ and relatively low breakdown strength. In the field of electrical engineering, polymer dielectrics are basically equal to the insulating polymers, meaning that the polymer matrix in polymer/polymer blended dielectrics usually is insulating polymers. According to the hysteresis loops (see Figure 7c), the insulating polymers can be divided into ferroelectric polymers, relaxor ferroelectric polymers, dipolar glass polymers, and linear dielectric polymers. Consequently, polymer/polymer blended dielectric composites consist of insulating polymer/insulating polymer blended dielectrics, insulating polymer/semiconductive polymer blended dielectrics and insulating polymer/conductive polymer blended dielectrics, as shown in Figure 7d.

#### 2.2.1 Insulating Polymer/Insulating Polymer Blended Dielectrics

##### Linear Polymer Dielectrics/Linear Polymer Blended Dielectrics

**Figure 8:**
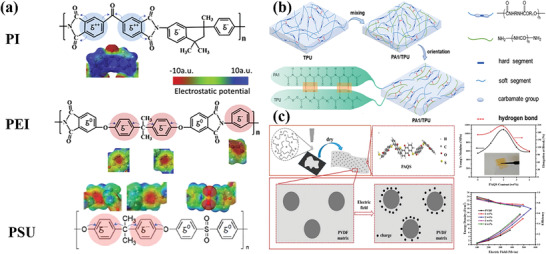
a) Schematic chemical structures of PI, polyetherimide, and poly(1,4‐phenylene ether‐sulfone). Reproduced with permission.^[^ ^14g^ ^]^ Copyright 2021, Elsevier. b) Schematic of hydrogen bond and orientation of molecule chains between TPU and PA1. Reproduced with permission.^[^ ^66^ ^]^ Copyright 2020, WILEY. c) The schematic diagrams of PAQS and various properties of PVDF/PAQS blend films. Reproduced with permission.^[^ ^83^ ^]^ Copyright 2020, Elsevier Science SA.

For dielectric polymers, the existence of voids and free volume will make the charge gain high energy in the process of movement, leading to the breakdown of polymers under lower electric fields. After different linear polymers are blended, tightly packed chain morphology will be gained via the electrostatic interaction, thereby reducing weak points.^[^ ^14^, ^59^ ^]^ The chemical structure diagram of PI, poly(ethylene imine) (PEI), and PSU is shown in **Figure** **8**a. PI has two positively charged phenyl groups, while PEI and PSU have three negatively charged phenyl groups. Zhang et al.^[^ ^14g^ ^]^ have tried to mix PEI and PI by solution casting method to improve the electrical properties of the composites. The 50%/50% PEI/PI polymer film can achieve a maximum breakdown strength of 650 MV m^−1^, which was about 45% higher than that of pure PI film. The energy density of the blend increased significantly to 8 J cm^−3^ (the energy density of pure PEI or pure PI < 5 J cm^−3^) at room temperature. At 200 °C, the breakdown strength of 50%/50% PEI/PI polymer film can be maintained at 450 MV m^−1^, which is 50% higher than that of pure PI film at the same temperature, and the energy density can be held at 1.4 J cm^−3^. PSU is a high‐temperature dielectric polymer with a *T*_g_ of 210 °C. Compared with PEI, PSU has a similar structure and exhibits the same negative charge on the phenyl group.^[^ ^14g^ ^]^ Further, Zhang et al.^[^ ^14g^ ^]^ have prepared PSU/PI blends, hoping to obtain higher breakdown strength. The results showed that the performance of PSU/PI was similar to that of PEI/PI. On the contrary, there is no strong electrostatic interaction between PSU and PEI, therefore, the PSU/PEI blend presented a low breakdown strength.

##### Linear Dielectric Polymer/Ferroelectric Polymer Blended Dielectrics

Although PVDF and other ferroelectric polymers have the high *Ɛ*_r_, their charge–discharge efficiency is suppressed due to the high loss, which seriously impedes the development of energy storage application. In order to alleviate the problem of high ferroelectric loss, the researchers have used linear dielectric polymers to reduce the dielectric loss and improve the efficiency of ferroelectric polymers,^[^ ^9^, ^60^, ^61^ ^]^ such as PMMA and polypropylene (PS). For example, by means of the good compatibility between ABS and PVDF, Yang et al.^[^ ^60^ ^]^ have blended ABS with PVDF through coagulation and hot‐press methods. Due to the strong interaction between ABS and PVDF, the ferroelectric domains of PVDF were weakened, the loss of the polymer was reduced and the efficiency was improved. Under an electric field of 400 MV m^−1^, an *U*_d_ of 5.7 J cm^−3^ and an efficiency of up to 82% were obtained when ABS/PVDF = 50/50 (the efficiency of pure PVDF is 55%). Meanwhile, ABS/PVDF showed better stability than that of pure PVDF in the cycle test.

As mentioned above, the bulky monomer chlorotrifluoroethylene (CTFE) was introduced into the main‐chain of PVDF to solve the problems of ferroelectric loss and conductivity of PVDF and improve its Ɛ_r_ and flexibility.^[^ ^62^ ^]^ In order to further improve the energy storage performance of P(VDF‐CTFE), Zhou et al.^[^ ^63^ ^]^ have mixed P(VDF‐CTFE) with PUA which is a linear dielectric material with high breakdown strength. The results indicated that the *Ɛ*_r_ of PUA/P(VDF‐CTFE) blend was lower than that of pure P(VDF‐CTFE), but the breakdown strength and efficiency were greatly improved.

It is well known that thermoplastic polyurethane (TPU) is an elastomer and may repair the defects in PVDF. Zheng et al.^[^ ^64^ ^]^ have reported that the TPU/PVDF blended polymer had higher breakdown strength than that of neat PVDF and TPU, keeping the original *Ɛ*_r_. Although TPU could increase the breakdown strength of the PVDF‐based composite, the high loading of TPU will deteriorate the energy storage property. Experiments have shown that when the TPU content exceeded 3 vol.%, serious phase separation decreased the breakdown strength of the blends. Besides the PVDF, linear TPU can also improve the energy storage performance of polyamides (PA) via forming hydrogen bonds. For PA, the *Ɛ*_r_ increased with the increase of hydrogen bond and dipole density.^[^ ^50^, ^65^ ^]^ Among all kinds of PA, the polyamide‐1 (PA1) contains the largest ‐HNCO‐ group density and dipole density, resulting in the maximal *Ɛ*_r_. Yuan et al.^[^ ^66^ ^]^ have prepared PA1/TPU composite films with different ratios by solution blended (see Figure 8b). The experimental results showed that adding PA1 significantly enhanced the Ɛ_r_ of TPU and reduced the dielectric loss of the polymer. When the content of PA1 is 1 wt%, the *Ɛ*_r_ of TPU can be increased sharply from 8 to 41. Moreover, the PA1/TPU composite film displayed good thermal stability and frequency stability.

The *Ɛ*_r_, dielectric loss and breakdown strength of the polymer matrix can be adjusted by blending another kind of polymer, however, the limitation of compatibility between polymers will lead to the decrease in breakdown strength and energy density. In order to improve the compatibility of the polymer matrix and polymer fillers, Yang et al.^[^ ^67^ ^]^ introduced PS‐block‐poly(methyl methacrylate) (PS‐b‐PMMA) into PVDF by solution casting method. It was found that the good compatibility between the PVDF matrix and PS‐b‐PMMA fillers is conducive to the improvement of the breakdown strength.^[^ ^68^ ^]^ When the content of PS‐b‐PMMA was 9%, the breakdown strength of the composite film reached 522 MV m^−1^ and the energy density was 10.1 J cm^−3^, which were much higher than that of pure PVDF. Xu et al.^[^ ^69^ ^]^ have used polydopamine as a solubilizer to coat the surface of PP, and then prepared a PP/PVDF composite film by a two‐step method of coagulation and hot pressing. Modified PP can not only increase the polarization of PVDF, but also apparently reduce the dielectric loss. The results showed that PP/PVDF composite film had compact structure and small crystallite size, which are beneficial to further improve the energy density and energy efficiency of the polymers. Among the PP/PVDF blends with different proportions, when PP:PVDF = 10:90, the best energy storage performance was obtained. Under the electric field of 350 MV/m, the energy storage density of the polymer was 12.1 J cm^−3^ with an efficiency of 69.3%.

High breakdown strength and low dielectric loss are very necessary for dielectric capacitors. When the dielectric loss is too high, the capacitor will generate a lot of heat during the charging and discharging process, which will eventually lead to dielectric failure and thermal runaway. For P(VDF‐TrFE‐CFE) terpolymer, the most shortcoming is the low breakdown strength. The linear polymer PMMA was added into P(VDF‐TrFE‐CFE) matrix via solution casting method by Liu et al.^[^ ^70^ ^]^ Compared with pure terpolymer P(VDF‐TrFE‐CFE), the PMMA/P(VDF‐TrFE‐CFE) blended polymer had lower dielectric loss and higher Young modulus. The greatest breakdown strength of the blended polymer reached 733 MV m^−1^, and the efficiency was maintained above 90% with great cycle stability.

The presence of CF_3_ groups breaks the symmetry of molecular chain, so the P(VDF‐HFP) has a lower crystallinity and a higher density under the same electric field than that of PVDF. However, P(VDF‐HFP) presents obvious polarization hysteresis under high electric field, leading to large energy loss.^[^ ^71^ ^]^ In contrast, the energy loss of PMMA is much lower, and the stability of the dielectric properties of PMMA as a function of frequency is good. Luo et al.^[^ ^72^ ^]^ have prepared PMMA/P(VDF‐HFP) blends via thermoforming methods. The good compatibility between P(VDF‐HFP) and PMMA decreased the dielectric loss of the composite film. Due to van der Waals forces, hydrogen bonds, and chain entanglement, the energy density of P(VDF‐HFP)‐based composite with 42.6 vol% PMMA was 11.2 J cm^−3^ and the efficiency was 85.8% under the electric field of 475 MV m^−1^.

##### Relaxor Ferroelectric Polymer/Ferroelectric Polymer Blended Dielectrics

Ferroelectric polymer with *β* phase has high *Ɛ*_r_ and breakdown strength, but it is prone to saturated polarization under low electric field, limiting the increase of the storage density. Jung et al.^[^ ^15j^ ^]^ have improved the energy density of dielectric materials by mixing relaxor ferroelectric polymer P(VDF‐TrFE‐CFE) with ordinary ferroelectric polymer P(VDF‐HFP). As the content of P(VDF‐TrFE‐CFE) increases, the *β* phase content of the blends decrease, leading to the increased non‐polar phase and the *Ɛ*_r_. When the ratio of P(VDF‐HFP) to P (VDF‐TrFE‐CFE) was 1:9, the energy storage density is up to 6.58 J cm^−3^ at an electric field of 250 MV m^−1^, which was significantly higher than that of the P(VDF‐TrFE‐CFE) ternary polymer. P(VDF‐TrFE‐CFE)/PVDF blends have been fabricated via solution casting by Zhang et al.^[^ ^15l^ ^]^ The experimental results showed that, compared with single component polymer, the blended polymer obtained higher *Ɛ*_r_ and electric displacement due to the existence of interfacial polarization effect. The energy density of the blended polymer was as high as 19.6 J cm^−3^.

According to the report, the *Ɛ*_r_ of P(VDF­ TrFE) polymer reached 9.6 at 1 kHz when the molecular composition of TrFE was more than 9%.^[^ ^73^ ^]^ Disappointingly, the P(VDF‐TrFE) polymer has a large remanent polarization and a large dielectric loss. In order to solve this issue, Zhang et al.^[^ ^15m^ ^]^ have blended P(VDF‐TrFE) with P(VDF‐HFP) in different ratios by solution casting method, and studied the influence of the spherulite size and crystallinity on the breakdown strength of the blended film. The study showed that when two polymers with similar Young's modulus were mixed, with the decrease of the spherulite size and crystallinity, the breakdown strength of the blend films could be greatly improved. When the blended ratio of P(VDF‐TrFE) and P(VDF‐HFP) was 5:5, the blend film obtained a breakdown electric field of 820 MV m^−1^ and an *U*_d_ of 23.8 J cm^−3^.

##### Dipolar Glass Polymer Dielectrics/Ferroelectric Polymer Blended Dielectrics

Aromatic polythiourea (ArPTU) is a dipolar glass polymer dielectrics with high breakdown electric field, high dielectric constant, and high discharge efficiency, which has attracted extensive attention of researchers.^[^ ^74^ ^]^ Unfortunately, ArPTU is brittle because of the presence of rigid aromatic groups, which is not suitable for preparing flexible dielectric films. Zhu et al.^[^ ^15n^ ^]^ have prepared ArPTU/P(VDF‐TrFE‐CTFE) composite film by solution blended and hot pressing. The results showed that the dielectric loss of polymer blends was reduced. In addition, an *U*_d_ of 19.2 J cm^−3^ with a charge–discharge efficiency of 85% under an electric field of 700 MV m^−1^ was achieved in the blended polymers. These improvements were inseparable from the improvement of the resistivity of the film and the change in the crystal structure.

PI is a glassy polymer with high breakdown strength, excellent thermal stability and mechanical properties.^[^ ^75^ ^]^ Frustratingly, the Ɛ_r_ of PI is in the range of 2.5–3.5, which is lower than other polymers used for energy storage.^[^ ^76^ ^]^ Polysulfone (PSF) has high Ɛ_r_ and high flexibility but low breakdown strength and thermal stability.^[^ ^77^ ^]^ Li et al.^[^ ^78^ ^]^ have prepared the PI/PSF composite film by using in‐situ polymerization and thermal imidization. Compared with pure PI and pure PSF, the obtained composite films presented the higher dielectric properties, thermal stability and mechanical properties. When the PSF content is 40 wt%, the composite film had a Ɛ_r_ of 6.4 and a breakdown strength of 152.2 MV/m. At the same time, it has a tensile strength of 94 MPa and a high T_g_. The dielectric properties and performance of various insulating polymer/insulating polymer blended dielectrics for capacitor applications are listed in **Table** **6**.

**Table 6:** The parameters of important insulating polymer/insulating polymer blended dielectrics for energy storage

Polymer A	Polymer B	Dielectric constant	Breakdown strength [MV m^−1^]	Energy storage density [J cm^−3^]	Efficiency	Ref.
ABS	PVDF	‐	400	5.7	82％	^[^ ^60^ ^]^
PEI	PI	3	600	8	‐	^[^ ^14g^ ^]^
TPU	PVDF	‐	537.8	10.36	‐	^[^ ^64^ ^]^
TPU	PA1	41	‐	‐	‐	^[^ ^66^ ^]^
PMMA	P(VDF‐TrFE‐CFE)	‐	733	10.672	90.2％	^[^ ^70^ ^]^
PS‐b‐PMMA	PVDF	‐	522	10.1	88％	^[^ ^67^ ^]^
PMMA	P(VDF‐HFP)	‐	476	11.2	85.8％	^[^ ^72^ ^]^
PMMA(45％)	PVDF	‐	640	17.7	73％	^[^ ^15i^ ^]^
PMMA(51％)	PVDF	‐	630	20.7	63％	^[^ ^15i^ ^]^
PTU	P(VDF‐CTFE)	‐	502	‐	75％	^[^ ^63^ ^]^
PP@polydopamine	PVDF	>12	350	12.1	69.3％	^[^ ^69^ ^]^
P(VDF‐HFP)	P(VDF‐TrFE‐CFE)	36	250	6.58	‐	^[^ ^15j^ ^]^
PVP	MD‐PVDF	‐	400	12	‐	^[^ ^79^ ^]^
P(VDF‐HFP)	PVDF	19.6	854	30.1	‐	^[^ ^15k^ ^]^
P(VDF‐TrFE‐CFE)	PVDF	30	640	19.6	‐	^[^ ^15l^ ^]^
P(VDF‐TrFE)	P(VDF‐HFP)	‐	820	23.8	63.8％	^[^ ^15m^ ^]^
ArPTU	P(VDF‐TrFE‐CTFE)	>10	707.2	19.2	85％	^[^ ^15n^ ^]^
ArPTU	P(VDF‐TrFE‐CFE)	30.02	407.57	22.06	72％	^[^ ^15o^ ^]^
PSF	PI	6.4	152.2	0.64	‐	^[^ ^78^ ^]^

#### 2.2.2 Insulating Polymer/Semiconductive Polymer Blended Dielectrics

Semiconductor refers to a material whose conductivity is between conductor and insulator at room temperature and has become an important raw material for flexible dielectric materials.^[^ ^80^ ^]^ In the blended system of insulating and semiconducting polymers, the semiconducting polymer can capture the injected charges from electrodes because of its high electron affinity to improve the charge polarization and reduce the leakage current of the blend.^[^ ^81^ ^]^ At present, semiconductor polymer mainly includes the poly(anthraquinone sulfide) (PAQS), polythiophene, copper phthalocyanine polymer, side‐chain polymers containing perylene bisimide or triphenylene, and so on.

The *Ɛ*_r_ of PAQS is 8 at room temperature, and PAQS is an organic semiconductor with a narrow energy band gap of 3 eV. Furthermore, when the morphology of PAQS in composites is nanoparticles, the existence of polar carbonyl bonds can enhance the mechanical properties of the polymer matrix.^[^ ^82^ ^]^ Du et al.^[^ ^83^ ^]^ have fabricated PAQS/PVDF composite (see Figure 8c). Without modifying the surface of PAQS nanoparticles, the PAQS is uniformly dispersed in the PVDF matrix. Compared with pure PVDF polymer, PVDF/PAQS blends exhibited a higher breakdown strength and energy density. Moreover, the mechanical properties of the composites were improved and the leakage current was decreased. When the content of PAQS was 2 wt%, a high energy density of 18 J cm^−3^ was achieved under the electric field of 560 MV m^−1^. Similar experiment results were found in the poly(1,4‐anthraquinone) (P14AQ)/PVDF blends by this group. The high breakdown strength of 614 MV m^−1^, excellent mechanical strength of ≈1.2 GPa of Youngs’ modulus and high energy density of 14.0 J cm^−3^ were obtained in 7 wt% P14AQ/PVDF composites.

Triphenylene side‐chain liquid crystalline polymer (PHT) has the advantages of high electron mobility, easy processing, good temperature stability, and solubility. Therefore, Qian et al.^[^ ^84^ ^]^ have put forward the idea of PHT/PVDF blends via solution mixing. In order to improve the compatibility between PVDF and PHT, the random copolymer PHT‐*co*‐P9F and block polymer PHT‐*b*‐P9F (P9F is poly[2‐(perfluorobutyl) ethyl methacrylate]) were fabricated, and the dielectric performance of PHT/PVDF, PHT‐*co*‐P9F/PVDF, and PHT‐*b*‐P9F/PVDF were investigated, respectively. The results showed that the PHT‐co‐P9F and PHT‐b‐P9F formed the smaller size of nanoparticles than that of PHT in the PVDF matrix due to hydrogen bonds between P9F and PVDF. The highest Ɛ_r_ of PHT/PVDF, PHT‐co‐P9F/PVDF, and PHT‐b‐P9F/PVDF were 17.2, 14.7, and 35.5 at 100 Hz, respectively. The main reason for this phenomenon is interface polarization.

Because the *π*‐electrons in the molecular chain are very delocalized, copper phthalocyanine oligomer (o‐CuPc) has a super *Ɛ*_r_ greater than 1000.^[^ ^85^ ^]^ Chen et al.^[^ ^86^ ^]^ have taken advantage of this feature to increase the Ɛ_r_ of polymer PI. Firstly, o‐CuPc was thermally dehydrated to synthesize a copper phthalocyanine anhydride oligomer (o‐CuPcA), then o‐CuPcA and amino‐capped polyamic acid were polycondensed, and finally thermally imidized at 300 °C to prepare CuPc‐PI copolymer. The highest *Ɛ*_r_ of 30 wt% o‐CuPcA polymer was 23.2, and the energy density was 0.55 J cm^−3^.

Poly(1, 6, 7, 12‐tetra‐chlorinated perylene‐*N*‐2‐aminoethyl acrylate‐*N*′dodecylamine‐3, 4, 9, 10‐tetracarboxylic bisimide) (PPDI) is a kind of typical N‐type semiconducting polymer, which possesses good solubility, thermal stability, chemical stability, and high electron affinity. The P(VDF‐TrFE‐CTFE) has a relatively high *Ɛ*_r_.^[^ ^81^ ^]^ Consequently, the semiconducting PPDI was selected as a filler and P(VDF‐TrFE‐CTFE) was used as the matrix, Qiao et al.^[^ ^81^ ^]^ have prepared the PPDI/P(VDF‐TrFE‐CTFE) blends. The *Ɛ*_r_ of the blends firstly increased and then decreased with the increase of PPDI filler. When the content of PPDI was 3 wt%, the maximal *Ɛ*_r_ was 33 at 1 Kz. The maximum energy density of the 4.5 wt% PPDI/PVTC blend polymer under an external electric field of 200 MV m^−1^ reached 4.42 J cm^−3^. Later, the effect of PPDI loading on the dielectric and energy storage performances of PPDI/PEI composites was investigated. The largest *Ɛ*_r_ of 7 at 1 kHz was detected in 4 wt% PPDI/PEI composite. At the temperature range from 25 °C to 150 °C, PPDI/PEI composite holds excellent temperature stability of dielectric properties. When the loading of PPDI was below 1 wt%, the breakdown strength of PPDI/PEI composite increased from 359 to 485 kV mm^−1^. The maximal energy density of 8.0 J cm^−3^ at 540 kV mm^−1^ with efficiency of 86% was attained in 1 wt% PPDI/PEI composite. In addition, the energy density of 5.12 J cm^−3^ at 500 kV mm^−1^ with the efficiency of 82% was obtained at 100 °C.

#### 2.2.3 Insulating Polymer/Conductive Polymer Blended Dielectrics

The addition of conductive fillers can greatly improve the dielectric constants of the nanocomposites. The nanocomposites can be used in electronic commerce, electromagnetic shielding, and other fields, which do not need to work in high‐voltage environment.^[^ ^87^ ^]^ When the conductive polymer is used as a filler, the *Ɛ*_r_ of polymer matrix can be significantly increased owing to interface polarization and micro‐capacitor. On the one hand, the strong interaction between the conductive polymer and the polymer matrix makes the mechanical properties of the composite material improve. On the other hand, the conductivity of the conductive polymer can be adjusted according to the requirements via changing the concentration of the conductive polymer.^[^ ^87^ ^]^ According to the percolation theory, as the content of conductive fillers increases to the percolation threshold (*φ*_c_), the *Ɛ*_r_ of the composite material will increase sharply. Hence, when *φ*_c_ is low, the composite material not only can obtain a largely enhanced *Ɛ*_r_, but also can keep good flexibility.^[^ ^81^, ^88^ ^]^

Polyaniline (PANI) with low cost and low elastic modulus can be blended with poly(vinyl alcohol), polyurethane, and other polymers to prepare high‐performance dielectric composite materials.^[^ ^89^ ^]^ Choosing PVDF as the polymer matrix and PANI as the filler, PANI/PVDF composite films were fabricated by Yuan et al.^[^ ^90^ ^]^ The PANI were evenly dispersed in the PVDF matrix due to the good compatibility between PVDF and PANI. When PANI content was 5 vol%, the *Ɛ*_r_ of PANI/PVDF composite was as high as 385 and the breakdown strength was 60 MV m^−1^.

In addition to the types of conductive polymers, the shape and size of conductive polymers have a great impact on the comprehensive energy storage performance of composites.^[^ ^91^ ^]^ Zhang et al.^[^ ^92^ ^]^ have used PPy nanoclips as a filler and P(VDF‐TrFE) as matrix to form the insulating polymer/conductive polymer blended dielectrics. P(VDF‐TrFE) presents high crystallinity and large polarization.^[^ ^93^ ^]^ The small size of the nanoclips can restrict the formation of excessively long conductive channels. When the PPy content was 0.8 wt%, the *Ɛ*_r_ of P(VDF‐TrFE)/PPy blends reached 1000 at room temperature and 2000 at a high temperature of 98 °C. Compared with conductive metal fillers, P(VDF‐TrFE)/PPy blends maintained a lower loss. P(VDF‐CTFE) has weak ferroelectric to paraelectric phase transition, which shows better dielectric properties. Zhang et al.^[^ ^94^ ^]^ have prepared nanocomposites with uniform structure by solution casting and hot pressing via using PPy nanoclips as filler and P(VDF‐CTFE) as matrix. The results demonstrated that P(VDF‐CTFE)/PPy composite material possessed high *Ɛ*_r_ and low percolation threshold. Specifically, when the content of PPy was 7 wt%, the *Ɛ*_r_ of the PPy‐P(VDF‐CTFE) composite material was 22 times larger than that of the pure P(VDF‐CTFE). A random coil PPy was prepared via an oxidative template assembly approach by Wang et al.^[^ ^95^ ^]^ Subsequently, PVDF/coil‐PPy composite films were fabricated by the traditional solution casting method. The PVDF/coil‐PPy composite film had good mechanical properties because the random coil PPy was dispersed uniformly in the PVDF matrix to reduce the structural defects. The interface interaction between the random coil PPy and PVDF can significantly improve the *T*_g_ of the materials. In addition, when the content of random coil PPy was more than 7 wt%, due to the formation of continuous network, obvious percolation transition can be seen in the composite. The discovery has an important impact on the development of electromagnetic interference and electromagnetic functional materials.

### 2.3 Layer Structured Polymer Dielectrics

The multilayer structure can suppress the extension of the electrical tree from the inside of the material and construct macroscopical interfacial polarization, resulting in improved breakdown strength of the material.^[^ ^96^ ^]^ More importantly, the dielectric properties of the material can be controlled by regulating the number of layers, layer thickness, and relative position of the layers of the multilayer structured polymer dielectrics.^[^ ^15^, ^97^ ^]^

#### 2.3.1 Two‐Layer All‐Organic Polymer Dielectrics

**Figure 9:**
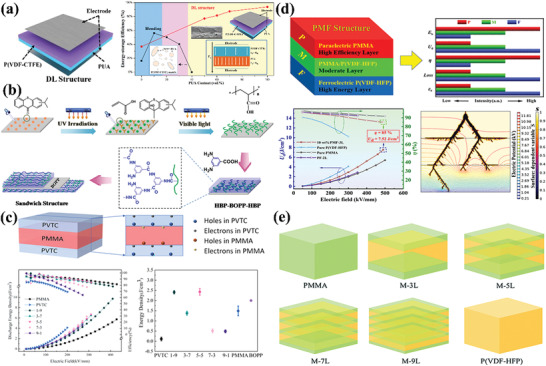
a) Schematic diagrams and illustration of the electric field distribution of DL composite film. Reproduced with permission.^[^ ^99^ ^]^ Copyright 2020, Elsevier Science. b) Schematic diagrams of preparing sandwich structured HBP‐BOPP‐HBP composite film. Reproduced with permission.^[^ ^15p^ ^]^ Copyright 2020, Wiley. c) Schematic diagrams of P(VDF‐TrFE‐CFE)/PMMA/P(VDF‐TrFE‐CFE), and various properties of P(VDF‐TrFE‐CFE)/PMMA/P(VDF‐TrFE‐CFE) with different contents. Reproduced with permission.^[^ ^15q^ ^]^ Copyright 2018, Institution of Engineering and Technology. d) Schematic illustration of the dielectric energy‐storage characteristics of the PMF structure. Reproduced with permission.^[^ ^108^ ^]^ Copyright 2021, American Chemical Society. e) Schematic illustration of the structures of the PMMA/P(VDF‐HFP) multilayer composites and the controls (pure PMMA and pure P(VDF‐HFP)), respectively. Reproduced with permission.^[^ ^113^ ^]^ Copyright 2020, Royal Society Chemistry.

The two‐layer structure film is an effective method to simultaneously obtain high *Ɛ*_r_ and breakdown strength via constructing the polarization layer and the insulating layer.^[^ ^97^, ^98^ ^]^ For example, Zhou et al.^[^ ^99^ ^]^ have prepared a P(VDF‐CTFE)/PUA double‐layer composite by a layer‐by‐layer casting method, as shown in **Figure** **9**a. Compared with the polymer blended composites, the double‐layer composite effectually avoided phase separation, and can fully combine the high dielectric properties of P(VDF‐CTFE) with the high energy storage efficiency of PUA. When the PUA content was 50%, the P(VDF‐CTFE)/PUA double‐layer composite material achieved the highest breakdown strength of 618 MV m^−1^. The *Ɛ*_r_ of P(VDF‐CTFE)/PUA double‐layer composites was similar to that of pure PUA, which is higher than that of pure P(VDF‐CTFE). Chen et al.^[^ ^14c^ ^]^ have synthesized the P(VDF‐TrFE‐CFE)/PI double‐layer composite by tape casting method in which P(VDF‐TrFE‐CFE) with nonferroelectric polarization was selected as top layer and linear polymer PI was used as the bottom layer. The results showed that the interface between P(VDF‐TrFE‐CFE) and PI was uniform and continuous, reducing the dielectric loss. Compared with P(VDF‐TrFE‐CFE) single‐layer film or PI single‐layer film, P(VDF‐TrFE‐CFE)/PI double‐layer film obtained higher *Ɛ*_r_, breakdown field strength, energy density, and energy efficiency. The aim of constantly adjusting the relative content of P (VDF‐TrFE‐CFE) was to harvest the best comprehensive energy storage performance. When the PI content was 50 vol%, an energy density of 9.6 J cm^−3^ with an efficiency of 58% was achieved for the double‐layer film.

#### 2.3.2 Three‐Layer Polymer Dielectrics

##### Symmetrical Three‐Layer Structure

Sandwich structures can integrate the characteristics of several different polymers, providing a new means for preparing high‐performance polymer dielectrics. In this section, the three layers of structured polymer dielectrics in which the top layer and the bottom layer are the same will be mainly described. Polycarbonate (PC) has high breakdown strength, but the Ɛ_r_ is low, only about 3.^[^ ^100^ ^]^ Mackey^[^ ^101^ ^]^ has selected PC as the top and bottom layers to improve the breakdown strength of the polymer, and PVDF with high dielectric constant as the intermediate layer to increase the *Ɛ*_r_ of the overall composite. The layered films were prepared by the method of micro/nanolayer coextrusion. The PC in the outer layer can play a role in resisting the entry of external charges into the PVDF layer, which makes the accumulation of charges in the PVDF less. When the content ratio of PC and PVDF was 50/50, the energy density of the sandwich structured film reached 11 J cm^−3^.

Epoxy resin has the advantages of low cost, easy processing, and good insulation performance, and can even reach nearly 100% charge–discharge efficiency under an electric field of 200 MV m^−1^. Consequently, Gao et al.^[^ ^102^ ^]^ have used epoxy resin as the top and bottom layers to improve the charge–discharge efficiency of the polymer dielectrics, and used PVDF as the intermediate layer to increase the *Ɛ*_r_. Epoxy/PVDF/epoxy polymer dielectrics with a sandwich structure by casting and hot pressing were formed. When the epoxy resin content was 75 vol%, epoxy/PVDF/epoxy composite films can achieve an *U*_d_ of 1.6 J cm^−3^ (three times that of BOPP) maintaining 96% charge–discharge efficiency under an electric field of 200 MV m^−1^.

Sandwich structured polymer dielectrics can be composed of different ferroelectric polymers to achieve high *U*_d_. Zhang et al.^[^ ^15s^ ^]^ have firstly prepared a fiber film layer of P(VDF‐TrFE‐CFE) with high *Ɛ*_r_ through an electrospinning method. Then, the PVDF/P(VDF‐TrFE‐CFE)/PVDF sandwich structured composite was fabricated by solution casting method and hot‐pressed method. Due to the excellent interface compatibility and the redistribution of the local electric field, the dielectric properties and breakdown strength of the PVDF/P(VDF‐TrFE‐CFE)/PVDF composites were effectively improved. The *Ɛ*_r_ of the composite was as high as 16 and the *E*_b_ reached 408 MV m^−1^, and the maximum *U*_d_ was up to 8.7 J cm^−3^. Wang et al.^[^ ^15t^ ^]^ have used PVDF as the top and bottom layers, and used P(VDF‐TrFE‐CTFE) as the intermediate layer to prepare all‐organic sandwich structured polymer dielectrics. Experiments showed that at a frequency of 1 kHz, when the content of P(VDF‐TrFE‐CTFE) was 45 vol%, the composite achieved the maximum *Ɛ*_r_ of 18.61. Under the same electric field, the *U*_d_ of the composite was increased with the increase of P(VDF‐TrFE‐CTFE) content. Finally, under an electric field of 600 MV m^−1^, a polymer containing 25 vol% P(VDF‐TrFE‐CTFE) can obtain a *U*_d_ as high as 20.86 J cm^−3^.

Sandwich structured polymer dielectrics do not have to be made of ferroelectric polymers. The enhanced energy storage density can also be achieved by sandwich structured composites composed of other polymers with excellent performance. BOPP is widely used in film capacitors due to its high breakdown strength, tensile strength, excellent thermal shrinkage, and tear resistance. However, BOPP cannot meet the requirements of high *U*_d_ for capacitors. The low *Ɛ*_r_ of BOPP makes the *U*_d_ of BOPP only about 2 J cm^−3^. Han et al.^[^ ^15p^ ^]^ have used BOPP with high breakdown strength as the intermediate layer, and chose hyper‐branched aromatic polyamide (HBP) as the top and bottom layers to boost the *Ɛ*_r_ of the composite, shown in Figure 9b. The thickness of the HBP layer was to command the energy storage capacity of the three layers of structured polymer dielectrics. When the thickness of the HBP layer was 2.06 µm, the *Ɛ*_r_ of the HBP‐BOPP‐HBP sandwich structure polymer was increased to 5.5, ensuring extremely low dielectric loss.

In addition, sandwich‐structured polymer dielectrics can be composed of linear dielectric and ferroelectric polymers. Using P(VDF‐TrFE‐CFE) as an intermediate layer and PMMA as the top and bottom layers to minimize the injection of electrode charges, Zhu et al.^[^ ^15q^ ^]^ have fabricated PMMA/P(VDF‐TrFE‐CFE)/PMMA sandwich structure film through a step‐by‐step solution casting method. PMMA as a charge blocking layer can greatly reduce the dielectric loss and leakage current density of the polymer dielectrics, and can improve the charge–discharge efficiency. Meanwhile, P(VDF‐TrFE‐CFE) can keep the *Ɛ*_r_ of the polymer dielectrics at a high level. By regulating the thickness of each layer, highest energy density of 7.03 J cm^−3^ with an efficiency of 78% was obtained under the electric field of 480 MV m^−1^. Feng et al.^[^ ^15r^ ^]^ have prepared the sandwich structured P(VDF‐TrFE‐CFE)/PMMA/P(VDF‐TrFE‐CFE) film by electrospinning method (see Figure 9c). By continuously adjusting the relative ratio of PMMA and P(VDF‐TrFE‐CFE), the energy storage performance of polymer dielectrics can be controlled for different requirements. As results, under the electric field of 400 MV m^−1^, the *U*_d_ of the polymer dielectrics with 10 vol% P(VDF‐TrFE‐CFE) content reached 9.7 J cm^−3^, and the charge–discharge efficiency is 78% at this time. Chen et al.^[^ ^103^ ^]^ have chosen P(VDF‐HFP) as the top and bottom layer, and PMMA as the intermediate layer to synthesize sandwich structure polymer dielectric by layer‐by‐layer solution casting method. Constantly adjusting the thickness of the layers can make the composites maintain a low dielectric loss and high *U*_d_ of 20 J cm^−3^ with a high energy efficiency of 80%.

Nowadays, the building up of extreme‐environment electronic devices, circuits, and systems entails high temperature‐capable electronic materials, among which dielectric polymers for high‐voltage capacitors are becoming the bottleneck. Hence, in order to meet practical application needs, it is essential to explore the dielectric capacitors with excellent energy density and stability under temperature fluctuations. PEI is an amorphous glassy polymer with a *T*_g_ of up to 217 °C and good temperature stability. At the same time, PEI has high breakdown strength and efficiency. The biggest disadvantage of PEI is low *Ɛ*_r_.^[^ ^105^ ^]^ Wang et al.^[^ ^14a^ ^]^ have produced positive‐sandwich structured composites, reverse‐sandwich structured composites, and single‐layered blends by casting layer by layer. Positive‐sandwich structured composites were composed of the top and bottom PEI layers, and the middle P(VDF‐TrFE‐CFE) layer. Because P(VDF‐TrFE‐CFE) has a high *Ɛ*_r_, when P(VDF‐TrFE‐CFE) is used as an intermediate layer, the *Ɛ*_r_ of the overall composite could be significantly improved. The outer layer PEI with excellent insulation performance could prevent a large amount of electrode charges from being injected into the intermediate layer. The breakdown field strength and energy efficiency of the composites were improved. PEI can also increase the working temperature of the composite because of its good temperature stability. Compared with polymer blends and the reverse‐sandwich structured composites, positive‐sandwich structured composites had higher *U*_d_ and were more stable. The positive‐sandwich structured composite with 15 vol% P(VDF‐TrFE‐CFE) achieved a breakdown electric field of 530 MV m^−1^, and obtained an *U*_d_ of 8 J cm^−3^ with a charge–discharge efficiency of 81% under this electric field. It is worth noting that it had excellent temperature stability of dielectric and energy storage properties from 25 to 100 °C.

To take advantage of each layer, some researchers have introduced a polymer blend layer into sandwich‐structured composites. Wei et al.^[^ ^106^ ^]^ have proposed a method of combining polymer blends and sandwich structures. First, P(VDF‐TrFE)/PVDF composites were prepared by blended P(VDF‐TrFE) and PVDF. Then the P(VDF‐TrFE)/PVDF composite was used as the middle layer, and PVDF was used as the top and bottom layers to form a sandwich structure PVDF‐P(VDF‐TrFE)/PVDF‐PVDF. The intermediate layer P(VDF‐TrFE)/PVDF composite film can increase the Ɛ_r_ of the composite. PVDF on the top and bottom layers can improve the breakdown strength of the composite. Studies showed that when the volume ratio of P(VDF‐TrFE) to PVDF was 30/70, the P(VDF‐TrFE)/PVDF composite film has an *U*_d_ as high as 23.6 J cm^−3^. The P(VDF‐TrFE)/PVDF intermediate layer with different volume ratios and the PVDF outer layer are combined to form a sandwich structure. The results showed that when the volume ratio of the P(VDF‐TrFE)/PVDF intermediate layer increased from 20% to 50%, the PVDF layer effectively resisted the injection of external charges and the dielectric loss was significantly reduced. Therefore, when the volume ratio of P(VDF‐TrFE) to PVDF was 50/50 and the content of the intermediate layer was 40% of the total, the sandwich structure film obtained an *U*_d_ of 20–24 J cm^−3^ with a charge–discharge efficiency of >65%.

By employing the interface coordination effect, Chen et al.^[^ ^107^ ^]^ have prepared a three‐layer polymer film in which PVDF/PMMA blends were used as the top and bottom layers and acrylic rubber dielectric elastomers (DE) were used as the middle layer. According to the research, under the electric field of 350 MV/m, the comprehensive energy storage performance of the three‐layer structure with 30 wt% PMMA was the best, and the *U*_d_ reached 15 J cm^−3^ with an efficiency of up to 76.5%. A good strategy to combine polymer blends and sandwich structures is to improve energy storage performance. Shen et al.^[^ ^15u^ ^]^ have adopted a simple modified polymer blended method to improve the efficiency and energy density of PVDF through the employment of nonpolar polypropylene (PP). The energy storage density of 8.1 J cm^−3^ and the efficiency of 75% was obtained at 300 kV mm^−1^ by further constructing a sandwich‐structured for PP/PVDF blend composites, compared with those of PVDF of only 4.7 J cm^−3^ and 38%, respectively.

##### Asymmetric Three‐Layer Structure

Different polymer layers in sandwich structures have been used to optimize *U*_d_ and charge–discharge efficiency. Shi et al.^[^ ^108^ ^]^ have designed a novel all‐organic polymer asymmetric trilayer structure film. The PMMA was used as the top layer to obtain a high discharge efficiency. The ferroelectric poly(vinylidene fluoride‐hexafluoropropylene) P(VDF‐HFP) was employed as the bottom layer to obtain a high *U*_d_. The PMMA/(P(VDF‐HFP) blends were used as the middle layer to homogenize the electric field inside the trilayer composites, turning out an obviously boosted breakdown strength and elevated *U*_d_. The experimental results showed that an efficiency as high as 85% and an outstanding *U*_d_ of 7.5 J cm^−3^ along with good cycling stability were simultaneously realized at an electric field of 490 kV mm^−1^ (see Figure 9d). Shi et al.^[^ ^109^ ^]^ have designed a unique asymmetric trilayer linear‐transition‐nonlinear (LTN) structure film. The nonlinear dielectric P(VDF‐HFP) layer offered high *U*_d_, and the linear dielectric PEI layer provided high discharge efficiency. The transition PEI/P(VDF‐HFP) layer could effectively homogenize the electric field distribution and increase breakdown strength of the film. In addition, the interfacial polarizations at the macro‐interface between adjacent layers and the micro‐interface inside the transition PEI/P(VDF‐HFP) layer further contributed to enhanced *U*_d_. The optimized LTN structure film achieved a high *U*_d_ of 12.15 J cm^−3^ and a high efficiency of 89.9%.

#### 2.3.3 Multilayer Polymer Dielectrics

Through using a multi‐layer co‐extrusion technology, multilayer polymer films can be prepared to improve the high temperature capability and *U*_d_ of the polymer dielectrics.^[^ ^110^ ^]^ Yin et al.^[^ ^111^ ^]^ have developed two kinds of multilayer structure PVDF‐based polymer capacitor via selecting polycarbonate (HTPC) and polysulfone (PSF) with high *T*_g_, respectively. The thermal stability of these two multi‐layer polymers were analyzed. Multi‐layer co‐extrusion method can ensure great uniformity between the layers. Because the *T*_g_ of PSF was higher than that of HTPC, the PSF/PVDF multilayer polymer exhibited a higher maximum operating temperature than the HTPC/PVDF multilayer polymer. When the temperature was lower than 170 °C, HTPC/PVDF multilayer polymer films and PSF/PVDF multilayer polymer films both obtained similar breakdown strength and energy loss. However, when the temperature was higher than 170 °C, the PSF/PVDF multilayer polymer film has stronger breakdown strength than that of HTPC/PVDF multilayer polymer film. The hysteresis loss of PSF/PVDF multilayer polymer increased with the increase in temperature, but the melt recrystallization process can effectively reduce the hysteresis loss of PSF/PVDF multilayer polymer.

Li et al.^[^ ^112^ ^]^ have fabricated two types of multilayer structure polymer films containing linear polymers (PC) with high breakdown strength and low loss and polar polymers (PVDF and nylon) with high Ɛ_r_. Compared with the pure polar polymer, the multilayer film produced via co‐extrusion technology can significantly reduce the injection of external charges, resulting in reducing the conduction of electrons and improving the breakdown strength. The multilayer structure PC/nylon films showed similar dielectric properties to PC/PVDF films. However, unlike wound multilayer PC/PVDF polymer films, these PC/nylon‐12 polymer films presented self‐healing behavior when holding under an electrical stress up to 2000 V (≈250 MV m^−1^), which provides a potential for next‐generation high *U*_d_ and high‐temperature film capacitors.

Combining PMMA with P(VDF‐HFP), Chen et al.^[^ ^113^ ^]^ have synthesized a series of alternating multilayer films with different layers via electrospinning (see Figure 9e). All multilayer composites were controlled at the same thickness (≈12 µm). Benefitting from the blocking effect of the multilayer structure and excellent insulation performance of PMMA, the breakdown strength and discharge efficiency of the multilayer composites were improved simultaneously. The composite with a 9‐layer structure exhibited the outstanding *U*_d_ of 25.3 J cm^−3^ and the highest charge–discharge efficiency of 84% at 728 KV mm^−1^. The experiment confirmed that for the multilayer composites with the same components, the blocking effect was enhanced with an increase in the number of layers, leading to a significant improvement in the breakdown strength (**Table** **7**).

**Table 7:** Summary of related properties of sandwich structure composite materials

Sandwich structured polymer	Dielectric constant	Breakdown strength [MV m^−1^]	Energy storage density [J cm^−3^]	Efficiency	Ref.
P(VDF‐CTFE)/PUA	5.7	618	‐	77％	^[^ ^99^ ^]^
P(VDF‐TrFE‐CFE)/PI	6.04	‐	9.6	58％	^[^ ^14c^ ^]^
HBP/BOPP/HBP	5.5	400	2.38	90％	^[^ ^15p^ ^]^
Epoxy/PVDF/Epoxy	‐	200	1.6	96％	^[^ ^102^ ^]^
PMMA/P(VDF‐TrFE‐CFE)/PMMA	5.2	480	7.03	78％	^[^ ^15q^ ^]^
P(VDF‐TrFE‐CFE)/PMMA/P(VDF‐TrFE‐CFE)	‐	407.96	9.7	78％	^[^ ^15r^ ^]^
P(VDF‐HFP)/PMMA(30 vol％)/P(VDF‐HFP)	‐	440	20.3	84％	^[^ ^103^ ^]^
P(VDF‐HFP)/PMMA(70 vol％)/P(VDF‐HFP)	‐	400	16.2	91％	^[^ ^103^ ^]^
PVDF/P(VDF‐TrFE‐CFE)/PVDF	16	408	8.7	60％	^[^ ^15s^ ^]^
PVDF/P(VDF‐TrFE‐CTFE)(45％)/PVDF	18.61	416	12.71	‐	^[^ ^15t^ ^]^
PVDF/P(VDF‐TrFE‐CTFE)(25％)/PVDF	12.06	660	20.86	62％	^[^ ^15t^ ^]^
PEI/P(VDF‐TrFE‐CTFE)/PEI	‐	530	8	81％	^[^ ^14a^ ^]^
PVDF/(P(VDF‐TrFE)/PVDF)/PVDF	>10	609	23.6	66.3％	^[^ ^106^ ^]^
(PVDF/PMMA)/DE/(PVDF/PMMA)	‐	350	15	76.5％	^[^ ^107^ ^]^
(PP/PVDF)/PVDF/(PP/PVDF)	‐	300	8.1	75％	^[^ ^15u^ ^]^
PMMA/(PMMA/P(VDF‐HFP))/P(VDF‐HFP)	‐	490	7.5	85％	^[^ ^108^ ^]^
PEI/(PEI/P(VDF‐HFP))/P(VDF‐HFP)	‐	535	12.15	89.9％	^[^ ^109^ ^]^
PMMA/P(VDF‐HFP) (9 layer)	‐	728	25.3	84％	^[^ ^113^ ^]^

## 3 Conclusion

Dielectric capacitors are well known for their high‐power density, stability, and long life, which endow the broad prospects in lots of applications. However, one of the biggest shortcomings of dielectric capacitors is the low discharge energy density (*U*_d_), which limits the miniaturization and intelligent applications in electronic and electrical devices. In recent years, all‐organic film dielectrics are attracting wide interest due to their advantages of high breakdown strength, high energy density, good flexibility, and mass production.^[^ ^114^ ^]^ The review summarizes the development strategies of all‐organic film dielectrics, including molecular structure designed dielectric polymers, dielectric polymer blends, and layer structured dielectric polymers. Based on the review, three aspects can be highlighted for future research avenues on all‐organic polymer dielectric films with excellent dielectric properties and energy storage:

### 3.1 Molecular Structure Design of Dielectric Polymers Containing Polar Groups

For the design of molecular structure, polar groups with high dipole moment (such as urea, thiourea, imide, sulfone, etc.) can be introduced into the main chain of polymer, and the dielectric constant of polymer dielectrics can be improved because of orientation polarization. However, some dipole polar groups on the main chain are not easy to rotate, which will lead to excessive dielectric loss and low dielectric constant. Therefore, it is proposed to add hydroxyl group, ionic group, sulfone group, cyano group, thiophene group, and other polar groups into the side chain of polymer to greatly enhance the dielectric constant and reduce the loss. Furthermore, the higher the dipole moment of the polar group and the smaller the volume of the group, the easier it is to rotate, which can more effectively increase the energy storage performance of the material. In addition, the dielectric properties of the polymer PVDF can be regulated by changing the crystal structure, crystal size, crystallinity, crystal domain based on chemical modification methods such as grafting, block, and copolymerization.

### 3.2 Polymers Blended Dielectrics

Blended is a good way to regulate the structure and properties of the polymer matrix by selecting the function of organic polymer filler. The predominant mechanical properties and energy storage performance are achieved in polymer blends via improving the interface compatibility of polymers. In particular, the advantages of dipolar glassy polymers are more obvious because high glass transition temperature can reduce the loss of electronic and ion conduction of the overall polymer blends.

### 3.3 Layers Structured Polymer Dielectrics

Constructing a sandwich structure containing a polarization polymer layer with high dielectric constant and an insulation polymer layer with high breakdown strength and low loss, the polymer films can endure high voltage. Furthermore, the extension of the electrical tree from the inside of the material is suppressed, thereby the breakdown strength and dielectric constant of the material are improved. More importantly, the desired dielectric properties can be obtained by changing the number of layers, layer thickness, or other factors of the composite material.

The energy density and energy efficiency of the capacitor need to be further enhanced, so that the dielectric capacitor can expand the application range. On the other hand, the improvement of the charge–discharge efficiency can also save resources and avoid energy waste. In addition, the temperature stability, frequency stability, cycle times, and other issues of capacitors should be solved urgently in order to be used in extreme environments.

In a word, there is still a long way to explore the application research of capacitor. The development trend of thin film capacitors includes the following directions:

#### 3.3.1 High Dielectric Constant and High Energy Density

The *U*_d_ of commercial BOPP and BOPET‐based film capacitors is lower than 3–5 J cm^−3^, which is difficult to meet the urgent needs of miniaturization and lightweight of electronic devices. Taking BOPP‐based capacitor as an example, the weight will reach 120 kg when the size of capacitor is 700 mm × 220 mm × 515 mm. If the dielectric constant of the dielectric film is increased by one time, the weight and volume both can be increased by ≈40% under the same applied electric field.^[^ ^14d^ ^]^ The challenge is to solve the inverse relationship between dielectric constant and breakdown electric field.

#### 3.3.2 Ultra‐Thin Dielectrics and Miniaturized Device

Reducing the thickness of dielectric film is to enhance the breakdown electric field of polymer dielectrics, which is very meaningful to increase the capacitance energy density and meet the demand of significantly increasing large capacitance film capacitors.^[^ ^115^ ^]^ However, ultra‐thin dielectric film demand very high requirements for processing technology, referring to increased internal defects and easily damaged. In industry, the extreme thickness of the dielectric film can reach ≈2 µm, while which is >5 µm in the lab.

#### 3.3.3 Withstanding Extreme Environments Such as High Temperature

Conventional thin film capacitor cannot work in high temperature and humidity environment for a long time. The suitable operating temperature and average humidity for film capacitors are −40–35 °C and <70%, respectively. However, in lots of application scenarios, the environmental condition is harsh including high temperature, high pressure, high humidity, etc.^[^ ^17^ ^]^ For example, to ensure the stable operation of electric vehicles, the cooling system is required because the temperature of the engine exceeds 140 °C, while the extreme temperature of the DC capacitor is 85 °C.

## Conflict of Interest

The authors declare no conflict of interest.
